# Phase space methods for non-linear analysis of pedalling forces in cycling

**DOI:** 10.1371/journal.pone.0198914

**Published:** 2019-04-18

**Authors:** Alexander Kunert, Marcel Ott, Thomas Reuter, Daniel Koska, Christian Maiwald

**Affiliations:** 1 Department of Medical Engineering and Electronics, ICM - Institute for Mechanical and Industrial Engineering, Chemnitz, Germany; 2 Department of Human Movement Science and Health, Chemnitz University of Technology, Chemnitz, Germany; Universite de Nantes, FRANCE

## Abstract

**Introduction:**

From the perspective of dynamic systems theory, stability and variability of biological signals are both understood as a functional adaptation to variable environmental conditions. In the present study, we examined whether this theoretical perspective is applicable to the pedalling movement in cycling. Non-linear measures were applied to analyse pedalling forces with varying levels of subjective load.

**Materials and methods:**

Ten subjects completed a 13-sector virtual terrain profile of 15 km total length on a roller trainer with varying degrees of virtual terrain inclination (resistance). The test was repeated two times with different instructions on how to alter the bikes gearing. During the experiment, pedalling force and heart rate were measured. Force-time curves were sequenced into single cycles, linearly interpolated in the time domain, and z-score normalised. The established time series was transferred into a two-dimensional phase space with limit cycle properties given the applied 25% phase shift. Different representations of the phase space attractor were calculated within each sector and used as non-linear measures assessing pedalling forces.

**Results and discussion:**

A contrast analysis showed that changes in pedalling load were strongly associated to changes in non-linear phase space attractor variables. For the subjects investigated in this study, this association was stronger than that between heart rate and resistance level. The results indicate systematic changes of the pedalling movement as an adaptive response to an externally determined increase in workload. Future research may utilise the findings from this study to investigate possible relationships between subjective measures of exhaustion, comfort, and discomfort with biomechanic characteristics of the pedalling movement and to evaluate connections with dynamic stability measures.

## Introduction

**Fig 1:**
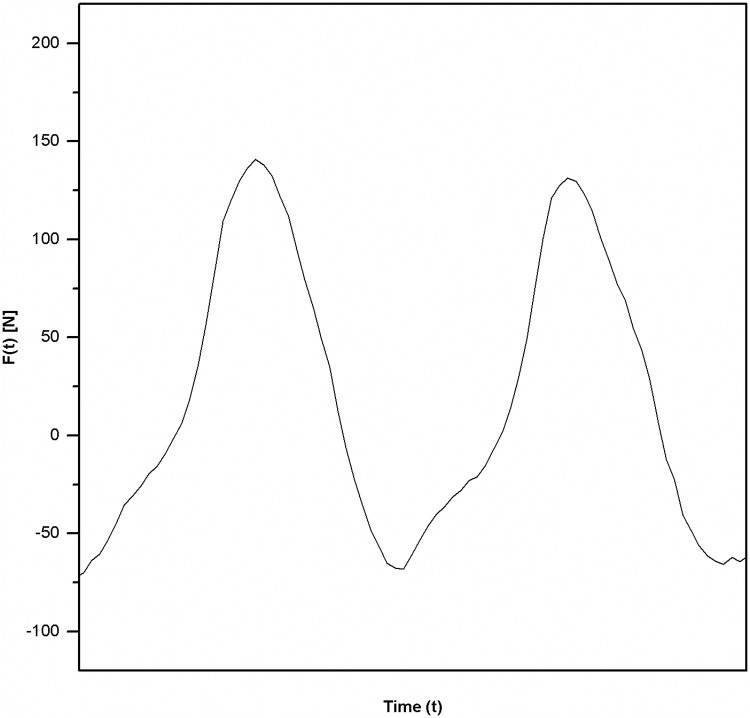
Typical pedal force course of the left leg during two pedalling cycles.

**Fig 2:**
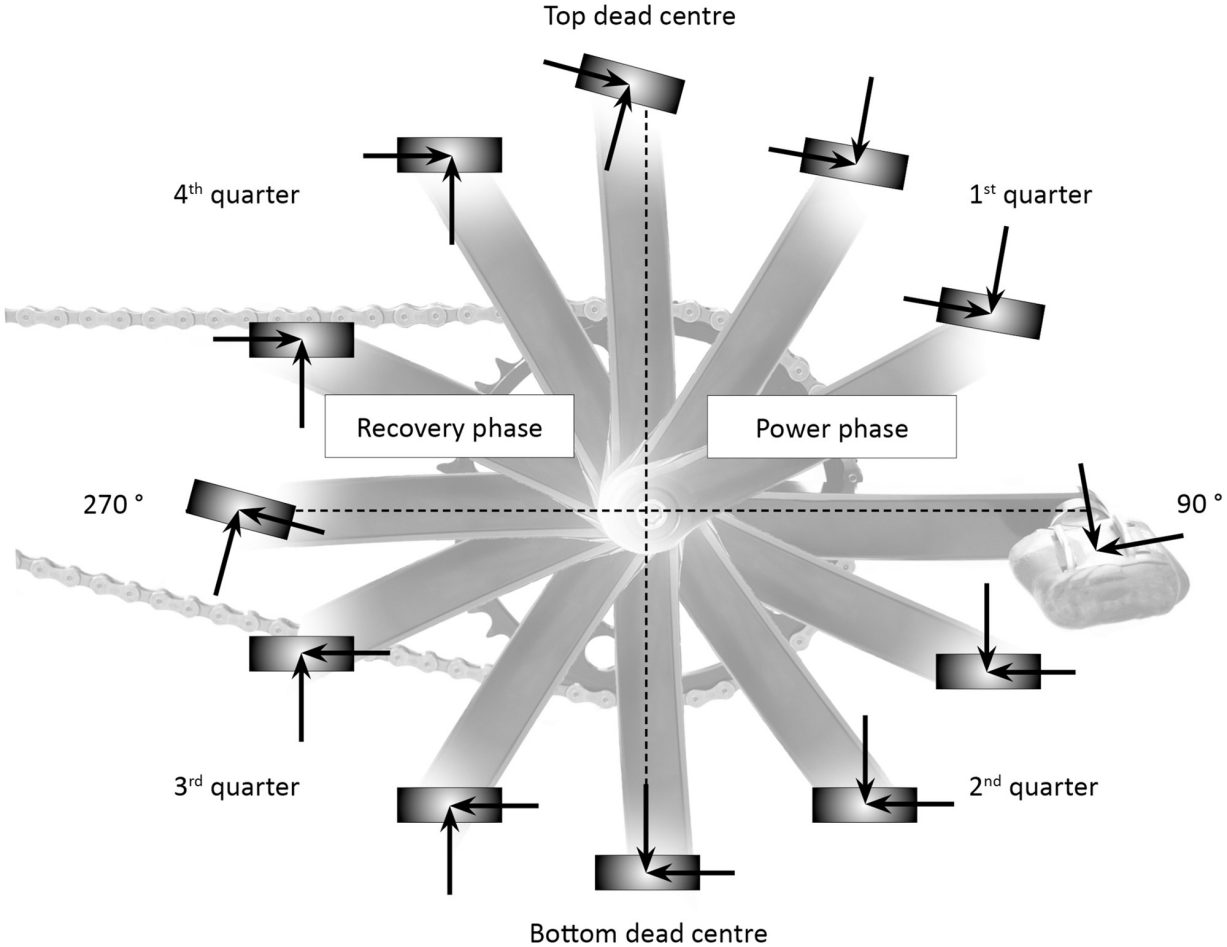
Phases of pedalling motion(own illustration based on Bini & Rossato [1]).

The physical properties of the pedalling movement in cycling have been a research subject in sports science for over 30 years. The kinetic aspects of pedalling during cycling can be comprehensively described and analysed using the time series of pedal forces across pedalling cycles. This tangential force-time curve shows the typical sinusoidal waveform depicted in Fig 1, which can be divided into individual movement phases and crank positions (Fig 2).

The pedalling movement can be analysed comprehensively using electromyographic and kinematic methods. Employing these methods, a solid basis of evidence has been established about the interaction and interdependencies of variables like pedal forces, pedalling efficiency, cadence, body position, expertise, pedalling asymmetry, and workload [2, 3]. Both the cadence and the force curve have been studied extensively with regard to the effects of different influencing variables (e.g. seat position, gradient, handlebar grip) on the efficiency of the pedalling movement [4–10], predominantly investigated on samples of competitive cyclists.

Kautz et al. [5] observed changes in tangential and normal torque during pedalling as a function of the workload. The authors found differences in the effective direction of the force vector and the pedal angle, but with a limited focus on the upstroke movement using clipless pedals (cleats). Hansen et al. [6] describe changes in peak power and range of the force curve within the pedalling cycles at constant power and cadence depending on the terrain inclination. Different angular accelerations are suspected as one reason for changes in power curves. Bertucci et al. [7] found little difference between uphill and level pedalling at the same cadence (80 rpm) and power (325W) in field tests. Emanuele and Denoth [11] compared level and uphill cycling concerning the freely chosen cadence and seat position. They observed lower cadences in combination with more upright body positions during uphill compared to level cycling.

Arkesteijn et al [12] tried to analyse changes in pedalling techniques due to different gradients on an adjustable treadmill. Three different gradients were tested while controlling the power output. Significant changes in pedalling forces were observed for the highest gradient of 8% in quadrants of downstroke and bottom dead centre.

Rossato et al. [10] analysed cyclists’ pedal forces and kinematics in 30-minute submaximal tests (60% and 80% of maximum power). Intervals of 10 minutes were performed at three cadences: freely chosen, 20% increased, and 20% lower than preferred. The authors found higher workloads resulting from lower freely chosen cadence and more effective recovery phase power (see Fig 2).

The cyclical characteristic of the force-time curves as well as the cyclic kinematic nature of the movement show strong parallels with gait and running movements. The variability and stability of time series in walking and running have been linked to pathologies, gait disturbances, (running) expertise, and other factors influencing the quality of motor regulation [13–16]. However, such hypotheses have rarely been investigated in research on pedalling and cycling movements.

The study by Warlop et al. [17] suggests that pedalling movement (similar to gait movement) has a non-linear temporal variability structure, and that metronome-controlled cadence leads to changes in the non-linear variability in comparison to a freely chosen cadence. Another study aimed to compare cadence variability with seating position on spinning bikes [18]. The study examined frequency-based measurements using rescaled range analysis (RRA) and power spectral density (PSD) to investigate the presence of long-term correlations, as well as standard deviation (SD) and coefficient of variation (CV) as linear variability measures. Both non-linear variability measures showed significant changes in fixed versus freely chosen cadence. Linear variability measures (SD, CV) were unable to detect any significant differences in the variability or stability of pedalling behavior.

In addition to the limited number of studies on non-linear measures of pedalling patterns and its functional correlates, pedalling behavior of non-athlete cyclists in non-competitive testing environments is largely unstudied. Due to the rapid social dissemination of e-bikes, pedalling motion is also receiving growing interest from a technological point of view [19]. The availability of crank- or hub-integrated mobile dynamometers offers the possibility of real-time monitoring of kinematic and kinetic pedalling parameters. From a scientific point of view, questions emerge regarding how this type of data can be used to describe and analyse motion patterns in cycling, especially in the everyday use of e-bikes. In the context of e-bikes, this translates to the hypothesis that the non-linear characteristics of the force-time-curves are associated with a preferred pedalling pattern. Preferred pedalling patterns may change if external stimuli (e. g. higher resistance through uphill cycling) are inflicted, and thus have an effect on non-linear measures of pedalling kinetics.

To our knowledge, ther are currently no studies available which have investigated whether any information on the cyclists’ workload can be associated to non-linear dynamic pedalling parameters. For new data sets it is recommended to use different analyses starting with visual inspectation with phase space methods [20]. The pedalling parameters should be used to drive the development of biosignal-controlled e-bike propulsion algorithms. Subsequently, our study aimed to investigate the possible relationship between individual adaptations of the pedalling movement in response to changes in external load and non-linear characteristics of pedalling forces.

## Materials and methods

### Subjects

From 19 healthy subjects measured initially, data from ten subjects could be included into the evaluated data set due to problems in data collection and data processing. All participants were informed orally and in writing about the purpose of the test and the basic procedure and gave written informed consent to their participation. The study was part of a larger study framework, which was approved by the Ethics Committee at Chemnitz University of Technology. Age, weight, and height (Table 1) were recorded and weekly workout volumes were categorised into cycling workouts and other sports activities.

**Table 1:** Summary of subjects’ anthropomtric data and workout volume.

parameter	unit	mean	SD
age	years	32.7	6.9
height	cm	178.4	7.9
mass	kg	75.5	10.4
activity	h/week	3.7	3.0

### Experimental protocol

**Fig 3:**
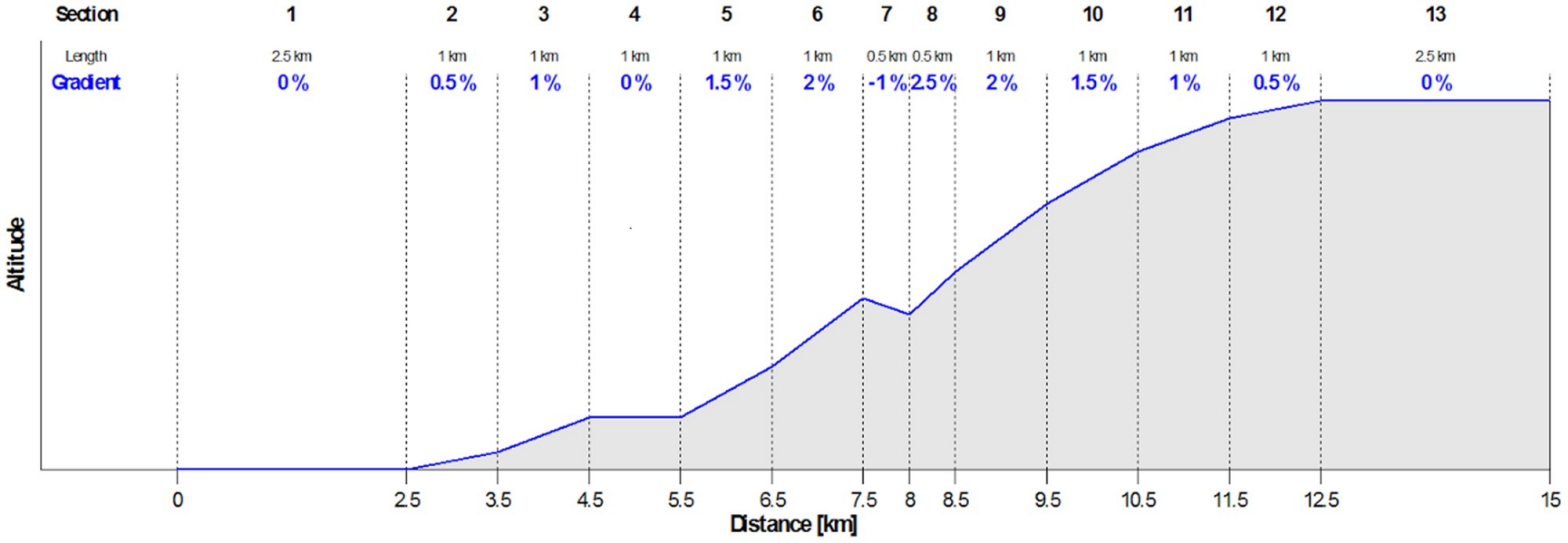
Terrain of the test scenario devided into 13 sections.

Participants were instructed to cycle along a virtual terrain profile on a mountain bike (Stein Bikes Mauna Loa 29AL) strapped to a roller trainer (Tacx Genius, tacx bv, Netherlands). In all measurements, participants wore their own running shoes, and platform pedals (Shimano Deore) were used to ensure comparable power transmission. The test protocol had 13 different sections with a total length of 15 km (Fig 3). Within this protocol the resistance force of the roller trainer was controlled by Tacx software (Tacx Trainer Software Version 4) depending on the simulated inclination. Simulation of higher gradients means a higher resistance force, lower or negative gradients are simulated by less resistance. Mass and height (75 kg, 180 cm) were set as constants across all subjects in the software protocol to ensure reproducible resistances. The participants were asked to choose their cadence freely. Since sections 1-3 and 12-15 were designed to be load-invariant and be used for protocol accommodation, only segments 4 through 11 were included in the data analysis.

Two sessions were carried out per subject, with different instructions on how to alter the bike’s gearing. In one session (NO SHIFT), subjects were asked to choose a preferred gear suitable for a range of terrain inclinations based on their subjective comfort during warm-up in simulated flat terrain. Subjects were instructed not to change their chosen gear for the remainder of the session. In the second session (SHIFT), no restrictions were given with regard to gearing, and subjects were allowed to use all 30 gears of the Shimano Deore shifters. Sessions were carried out in randomised order across subjects. Subjects were blinded regarding the length and inclination pattern of the virtual terrain, and that the identical terrain profile was used across all subjects and sessions. The saddle height selected in the first test was documented and reproduced during the second session to ensure a comparable seating position. The handlebar position was constant for all subjects. The two sessions were conducted no less than seven and no more than 21 days apart to ensure proper rest and no physiological or cognitive carry-over effects.

### Data collection

Raw torque data was recorded at a sampling rate of 64 Hz using a crank powermeter (Stages, Stages Inc., USA) on the left pedal arm (175 mm length, Shimano XT). Tangential pedalling force was calculated from raw data. It was synced to heart rate recordings using a BerryKing (bestbeans UG, Germany) chest strap. All measurements were transferred to a National Instruments myRio control computer and recorded using LabView (National Instruments, USA). Mean power [W], mean speed [km/h], mean cadence [1/min] and overall test time [min] were taken from the measurement protocol of the roller trainer software (Tacx Trainer Software Version 4) to get an overview of the test conditions. Mean relative power[W/kg] was calculated from the mean power, a taking into account the subject’s body mass.

### Data analysis

For each subject and session, the recorded data were devided into segments for each terrain sector 4-11 (see Fig 3). Force-time curves were sequenced into single pedalling cycles. Since to our knowledge there is no reliable algorithm for sequencing the force-time curves into single cycles, the original time series was dissected using the zero crossings with positive curve gradients. The data snippets for each force-time-cycle were linearly interpolated in the time domain to 100 values to normalise effects of changing cadence and reassembled into one time series per segment. The force values were z-standardised, based on the arithmetic mean and the standard deviation of the segment:

$$\begin{array}{c} \bar{z_{i}}=\frac{x_{i}-\bar{x}}{s} \end{array}$$

A two-dimensional phase space was created using Takens’ theorem [21].

$$\begin{array}{c} Z=\{z_{1},\;z_{2},\;z_{3},\ldots \;z_{i}\} \end{array}$$

The resulting phase space contains pairwise *τ*-delayed time-series points of the original time series, defined as:

$$\begin{array}{c} P=(z_{\left(t\right)};\;z_{\left(t+\tau \right)}) \end{array}$$

**Fig 4:**
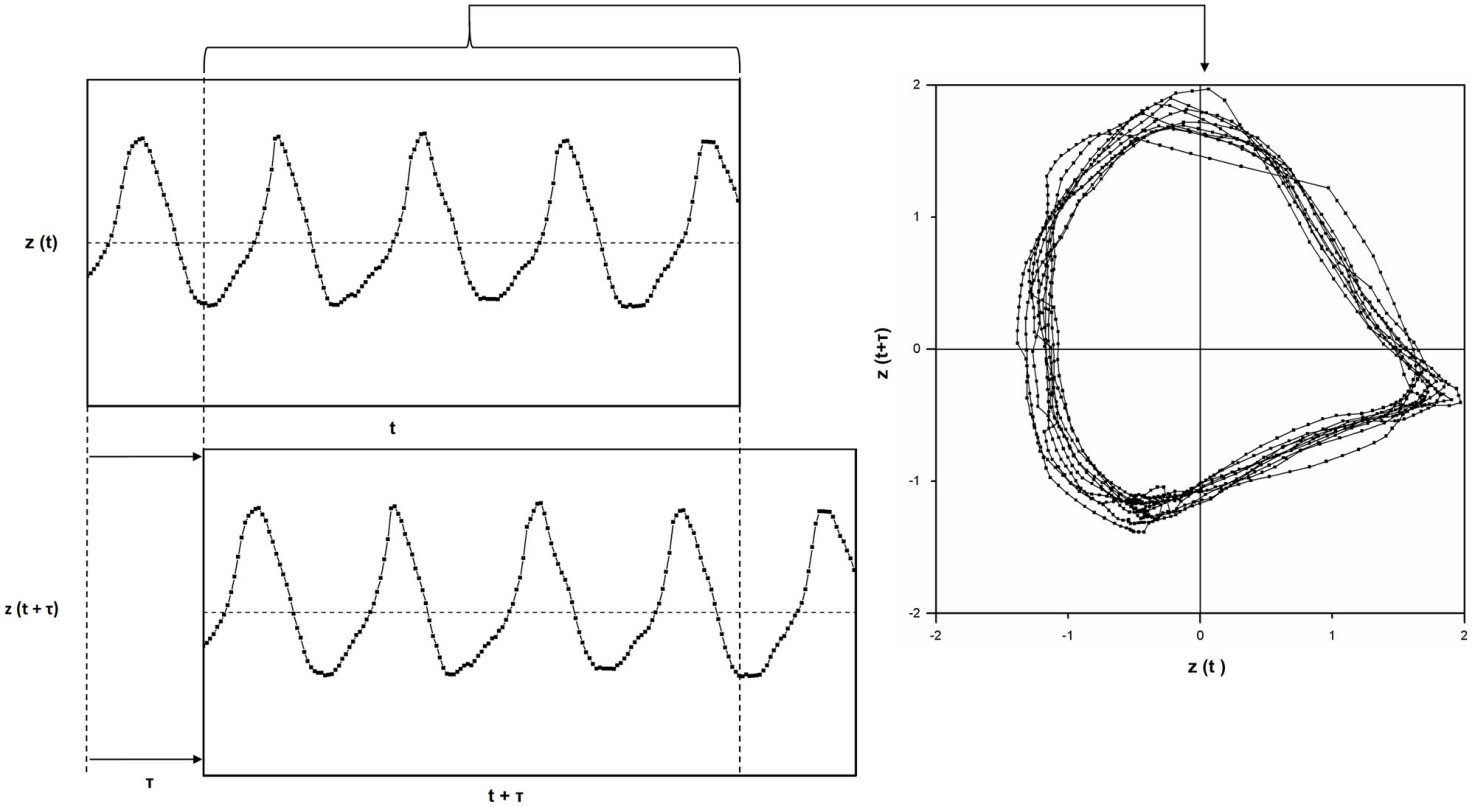
Sinusoidal curve of the z-normalised force(left) and associated phase plot (right)—Transcription into the phasespace was done according to Takens’ theorem (*τ* = 0.25 cycles).

Different point clouds were unfolded depending on the phase shift *τ*. For *τ* = 0.25 cycles, the waveform of the force-time curves evolved into a limit-cycle phase space arrangement [20] (Fig 4).

The following established measures were adapted to quantify phase-space characteristics we found in our data and to develop new non-linear measures:

#### Center of Levenberg-Marquardt Circle (L_*R*_)

**Fig 5:**
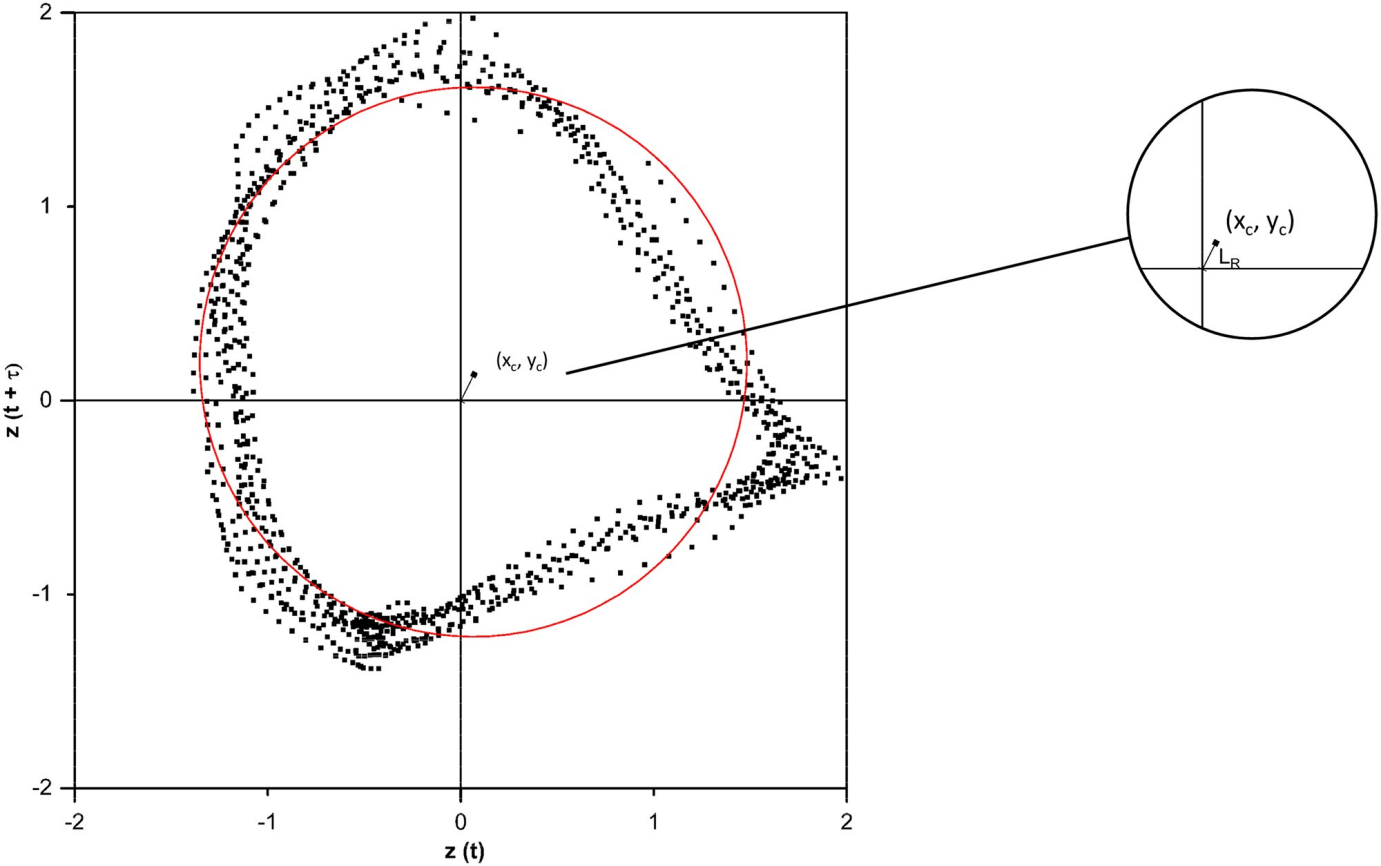
L_*R*_ was calculated as length of the vector between origin and the center of Levenberg-Marquardt-Circle.

The position of the circle-like point cloud in the phase space can be approximated by a regression circle. The resulting non-linear compensation problem is solved using the Levenberg-Marquardt algorithm [22] to achieve the best possible non-linear regression. If the center of the circle (x_*c*_, y_*c*_) deviates from the arithmetic center of the point cloud (coordinate origin), the length of the vector $L_{R}=\;\sqrt{x_{c}^{2}+y_{c}^{2}}$ between the two centers describes the outcome variable in the analysed terrain segment (Fig 5). The *L*_*R*_ value was calculated using R Package “conicfit” [23].

#### Componentwise median (M_*c*_)

M_*c*_ is calculated as the pair of unidimensional median coordinates of the point cloud in phase space, with the median calculated independently for each of the two dimensions (*M*_*c*_ = [*MED*(*x*_*i*_), *MED*(*y*_*i*_)]).

#### Geometric median (L1 median: M_*L*1_)

The geometric median (also called L1 median) is used as a robust indicator of central position of multivariate samples. In the current study, M_*L*1_ was computed using the R-package “GMedian” [24].

#### Revolution Time Coefficient of Variation (RevTimeCV)

RevTimeCV represents the linear variability of the pedalling cycles. It is calculated according to a coefficient of variation as the ratio between standard deviation (s) and arithmetic mean ($\bar{x}$) of the revolution times of all cycles within each terrain segment, and given as a percent value.

$$\begin{array}{c} RevTimeCV=\frac{s_{RevTime}}{{\bar{x}}_{RevTime}}\times 100 \end{array}$$

Additionally, the mean heart rate (HR) was calculated over all heart rate samples of each terrain segment, with heart rates sampled at 1 Hz.

### Statistical analysis

The z-standardisation and sequencing of the force signals, the calculation of L_*R*_, M_*c*_, M_*L*1_ and RevTimeCV, as well as their statistical evaluation were carried out using R [25]. In addition to exploratory analyses using boxplots, a contrast analysis of the outcome variables (see the previous paragraph) was carried out across the terrain segments. Intraindividual average values of each variable were used for this procedure, and it was hypothesised that these intrasubject averages of the outcome variables would change proportionally to resistance force or terrain inclination. Hence, the z-standardised terrain inclinations of sectors 4-11 were used as lambda weights. The prerequisites of the contrast analysis were tested using Shapiro-Wilk’s test for normality and Bartlett’s test for homogeneity of variances. Contrast analyses were performed for all non-linear measures (L_*R*_, M_*c*_, M_*L*1_), RevTimeCV and HR. As a result of the contrast analysis, the relationship between the segment inclination pattern and the pattern of the outcome variable is given in the form of an effect size parameter (Hedge’s g [26]). *α* = 0.01 was chosen as the level of statistical significance to ensure a sufficient test power of at least 0.8 despite the small sample size of ten subjects.

## Results

The overall results (section 1 to section 15) of the test conditions SHIFT and NO SHIFT are shown in Tables 2 and 3.

**Table 2:** Overall results of the SHIFT condtion: Arithmetic mean and standard deviation (SD).

parameter	unit	mean	SD
mean power	W	96.7	33.8
relative mean power	W/kg	1.3	0.4
mean speed	km/h	21.8	2.3
mean cadence	1/min	76.1	9.2
overall test time	min	42.1	4.7

**Table 3:** Overall results of the NO SHIFT conditon: Arithmetic mean and standard deviation (SD).

parameter	unit	mean	SD
mean power	W	68.3	25.9
relative mean power	W/kg	0.9	0.3
mean speed	km/h	19.9	2.3
mean cadence	1/min	78.4	7.4
overall test time	min	45.6	5.9

The following box plots contain the results of the outcome variables. The two sessions (SHIFT vs. NO SHIFT) are depicted next to each other in separate box plots to illustrate pattern changes between sessions.

**Fig 6:**
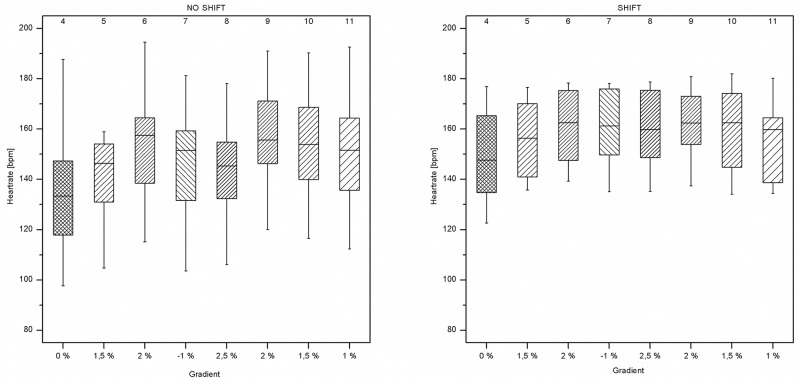
Boxplots of HR. Each box represents within-subject average HR values across all subjects within the same sector. The eight boxes for each plot represent the data from the sectors 4 to 11. The x-axes show the simualted terrain inclinations for each sector.

Heart rates varied between sectors and changes in heart rate were closely related to changes in terrain gradient in NO SHIFT. Much less variation in heart rate was visible in SHIFT (Fig 6).

**Fig 7:**
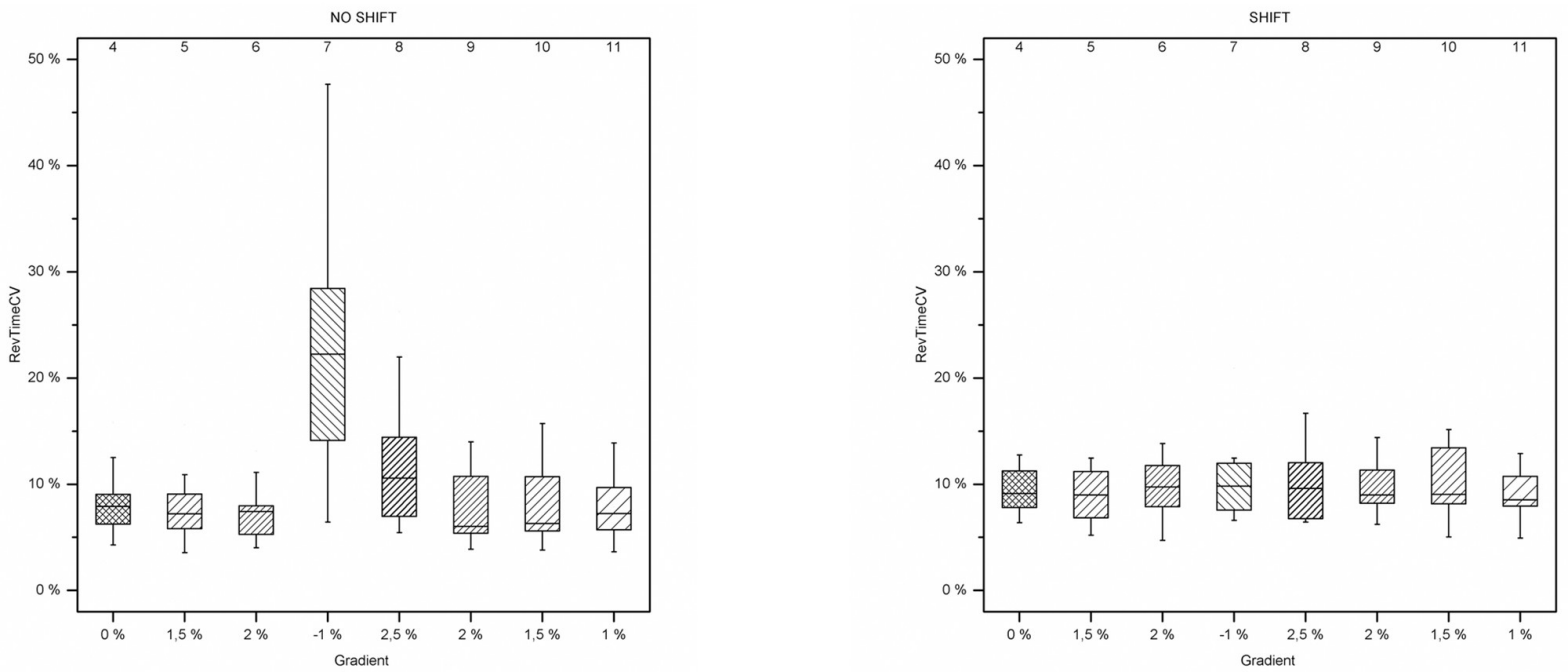
Boxplots of RevTimeCV. Each box represents RevTimeCV values across all subjects within the same sector. The eight boxes for each plot represent the data from the sectors 4 to 11. The x-axes show the terrain inclinations for each sector.

No association with the terrain inclination was found for RevTimeCV (Fig 7). Pedalling variance increased in NO SHIFT during the downhill and the following uphill phases, but remained largely constant under all other terrain conditions, and across sessions.

**Fig 8:**
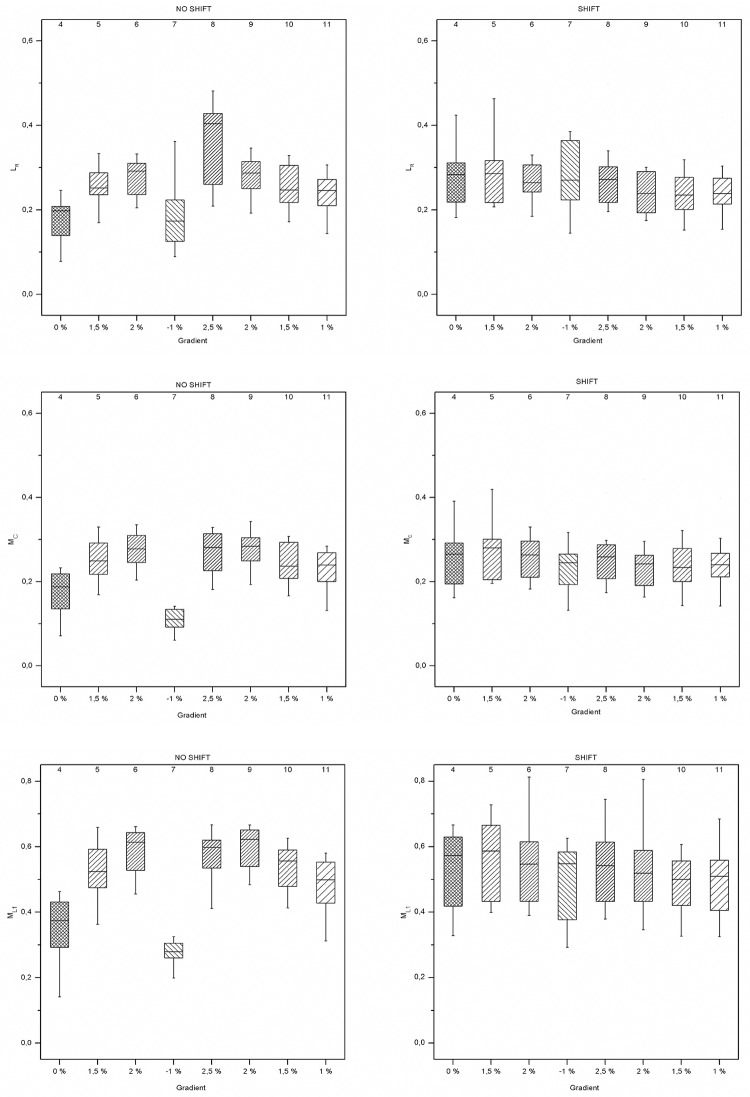
Boxplots of phase-space variables *L*_*R*_, *M*_*c*_, and *M*_*L*_1. Each box represents the specific non-linear measure across all subjects within the same sector. The eight boxes for each plot represent the data from the sectors 4 to 11. The x-axes show the terrain inclinations for each sector.

The following boxplots (Fig 8) depict the patterns in the non-linear variability measures. L_*R*_, M_*c*_, and M_*L*1_ are closely related to terrain inclination in NO SHIFT only. For SHIFT, none of the variables indicate a close relationship to terrain inclination.

The results of the contrast analysis are given in Table 4. For NO SHIFT, all dependent variables were closely related to terrain inclination. Highly significant associations (*p* < 0.001) were found for the terrain inclination and magnitude of L_*R*_, M_*c*_, M_*L*1_, and HR. For SHIFT, only HR was found to vary in a similar pattern compared to NO SHIFT (*p* = 0.009). RevTimeCV did not reveal any association with the terrain inclination.

**Table 4:** Summary of the contrast analysis results depending on the gradient pattern.

Condition	Parameter	HR	LR	Mc	ML1	RevTimeCV
SHIFT	t	2.905	0.028	1.500	1.538	-0.453
	p	0.009	0.489	0.084	0.079	0.669
	g	0.919	0.009	0.474	0.486	-0.143
NO SHIFT	t	5.719	5.632	7.426	11.714	1.188
	p	< 0.001	< 0.001	< 0.001	< 0.001	0.133
	g	1.808	1.781	2.348	3.704	0.376

t: t-value of the contrast analysis

p: statistical significance of the t-value

g: effect size (Hedge’s g) of the t-value

## Discussion

The results of our study indicate systematic changes in heart rate and pedalling forces as an adaptive response to increased physiological stress, caused in response to changed resistance of the roller trainer simulating different terrain inclinations. A contrast analysis revealed the expected increase in heart rate [27, 28], which resembles the simulated terrain pattern (Table 4). Furthermore, the contrast analysis showed that the heart rates were more closely associated with terrain inclinations in the NO SHIFT condition compared to SHIFT. The magnitude of adaptation varied between individuals, and presumably depends on the state of training and motivation, as well as cycling expertise. Conclusions on individual demands have to be made in follow-up studies in connection with endurance capacity tests to generalise the statements.

Changes in pedalling force have already been investigated under various influencing factors, such as cadence, saddle position, fatigue, and workload [10, 12, 29, 30]. The present study quantified significant adjustments to the distribution of the pedalling force. According to the result of the contrast analysis, the magnitude of the changes in the non-linear measures allow preadictions about the current terrain inclination which was simulated by changing the workload. However, the need for an individual reference measurement is not fundamentally excluded.

These observations are only valid for the NO SHIFT condition. During SHIFT, adaptations of pedalling dynamics—represented by non-linear measures L_*R*_, M_*C*_, and M_*L*1_—largely disappeared, since the additional degree of freedom related to gears can be used to alter the mode of power delivery. In NO SHIFT, the fixed gearing did not allow any type of adjustment in power delivery other than changing pedal forces to maintain cadence. Thus, every increase in power demand has to be accompanied by increases in pedalling forces to prevent the cadence from dropping substantially. This in turn seems to have triggered the changes in non-linear pedalling characteristics during NO SHIFT.

The subjects in this study were highly heterogenous with respect to expertise in cycling, individual fitness level, and age. Since we found homogenous results across many variables, the results appear to be transferable to a broader range of cyclists. However, a generalisation requires further investigation with larger samples and more realistic conditions in a field test.

Previous investigations on non-linear variability of pedalling were based on evaluations of cadence and cycle duration [17, 18]. The evaluation of the pedalling force in phase space is a methodological innovation in analysing biomechanic data obtained during cycling. It represents a non-linear interpretation of pedalling forces. Interlinks to established stability measures has to be discussed in following investigations. The applied normalisation and interpolation procedures eliminate differences in cadence and force amplitudes between subjects and across sectors. The remaining differences in phase space were caused merely by the geometric “shape” of the individual force-time series, and especially their evolvement over time across several cycles.

**Fig 9:**
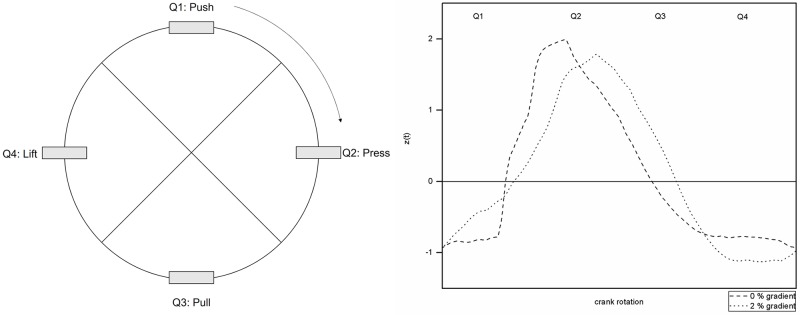
Schematic representation of one pedalling cycle divided into four quadrants. The figure on the right demonstrates qualitive differences in tangential pedal force development over the course of one pedalling cycle depending on terrain inclination. The figure on the left defines the four quadrants of one cycle.

To further interpret changes in non-linear variables with terrain inclination, we illustrate the progression of the normalised force-time curves across one cycle, divided into four quadrants. Each quadrant can be assigned a characteristic segment of this curve (Fig 9).

Changing resistance force of the roller trainer leads to different progressions of pedal forces, and causes shifts in the curves’ time-domain. These progressions of the normalised force time curves are illustrated in Fig 9 using data from a typical subject. Pattern changes in pedalling forces were observed as a rapid adjustments across only few cycles. The manifestations of change are diverse, possibly caused by dissimilar motor compensation and cycling experience. Typical change characteristics associated with increasing workloads are:

- Shift of the force maximum within Q2
- Prolongation of the downstroke phase with forces above the mean
- Increased linear variability of the forces in the area of the local minimum

Technologically, the measurement methods are based on a torsion measurement on the left crank arm. In view of the practical requirements for controlling propulsion systems in e-bikes, it is necessary to apply methods appropriate for use in the field. Nevertheless, it is necessary to investigate whether the described effects are observable for both legs in side sychronous measurements. The commercially available and mass-produced measuring instruments are not comparable with pure laboratory measuring instruments in terms of accuracy of the force measurement and sampling rate [29, 31]. For a mean cadence of 77.1 (+/- 8.0) cycles/min the used sample rate of 64 Hz provides a raw database of 49.81 measured values per cycle. Thus, sufficiently accurate measured values for temporal interpolation to 100 values per revolution were available. Additional evidence in field tests must be provided to evaluate the influence of other external disturbances (e.g. rolling and air resistance, traffic, uneveness of the ground) on the sensitivity of the non-linear quantities L_*R*_, M_*c*,_ and M_*L*1_. In compliance to the observations of Arkesteijn et al. [12] the simulation of uphill cycling by higher resistance of a roller trainer should be suitable for gradients less than 4%. Nevertheless, to rule out cross effects, the results should be verified in further studies, preferably in field studies with more realistic test conditions.

## Conclusions

In cycling, an externally determined stress due to larger workloads is characterised by systematic changes in the pedalling forces. Higher values for the measured variables represent characteristic changes in the shape of the force curve which occur depending the resistance level. Conscious avoidance of excessive stress by using the gears leads to seemingly more stable pedal forces. Prospectively, the individual distinction of different stress levels should be accessible based on non-linear measures of dynamic parameters. Therefore, a generalisability of the results has to be demonstrated by larger samples in further investigations. Further, it has to be proven that the methodology is suitable even for shorter observation periods. These investigations are necessary to provide the scientific basis for a real-time control algorithm of e-bike drives. For this, sliding window algorithms have to be developed and may be able to verify supposed connections between the discussed innovative non-linear measures and established stability measures.
